# Laser-Induced Transfer of Noble Metal Nanodots with Femtosecond Laser-Interference Processing

**DOI:** 10.3390/nano11020305

**Published:** 2021-01-25

**Authors:** Yoshiki Nakata, Koji Tsubakimoto, Noriaki Miyanaga, Aiko Narazaki, Tatsuya Shoji, Yasuyuki Tsuboi

**Affiliations:** 1 Institute of Laser Engineering, Osaka University, 2-6 Yamadaoka, Suita, Osaka 565-0871, Japan; tsubaki@ile.osaka-u.ac.jp; 2 Institute for Laser Technology, 1-8-4 Utsubo-honmachi, Nishi-ku, Osaka 550-0004, Japan; miyanaga@ilt.or.jp; 3 National Institute of Advanced Industrial Science and Technology, Central 2, Umezono 1-1-1, Tsukuba, Ibaraki 305-8568, Japan; narazaki-aiko@aist.go.jp; 4 Faculty of Science, Kanagawa University, 2946, Tsuchiya, Hiratsuka, Kanagawa 259-1293, Japan; t-shoji@kanagawa-u.ac.jp; 5 Graduate School of Science, Osaka City University, 3-3-138 Sugimoto Sumiyoshi-ku, Osaka 558-8585, Japan; twoboys@sci.osaka-cu.ac.jp

**Keywords:** interference laser processing, laser-induced dot transfer, laser-induced forward transfer, nanodot, nanoparticle, array, femtosecond laser, solid–liquid–solid mechanism, Pt

## Abstract

Noble metal nanodots have been applied to plasmonic devices, catalysts, and highly sensitive detection in bioinstruments. We have been studying the fabrications of them through a laser-induced dot transfer (LIDT) technique, a type of laser-induced forward transfer (LIFT), in which nanodots several hundred nm in diameter are produced via a solid–liquid–solid (SLS) mechanism. In the previous study, an interference laser processing technique was applied to LIDT, and aligned Au nanodots were successfully deposited onto an acceptor substrate in a single shot of femtosecond laser irradiation. In the present experiment, Pt thin film was applied to this technique, and the deposited nanodots were measured by scanning electron microscopy (SEM) and compared with the Au nanodots. A typical nanodot had a roundness $f_{r}=0.98$ and circularity $f_{circ}=0.90$. Compared to the previous experiment using Au thin film, the size distribution was more diffuse, and it was difficult to see the periodic alignment of the nanodots in the parameter range of this experiment. This method is promising as a method for producing large quantities of Pt particles with diameters of several hundred nm.

## 1. Introduction

**Figure 1:**
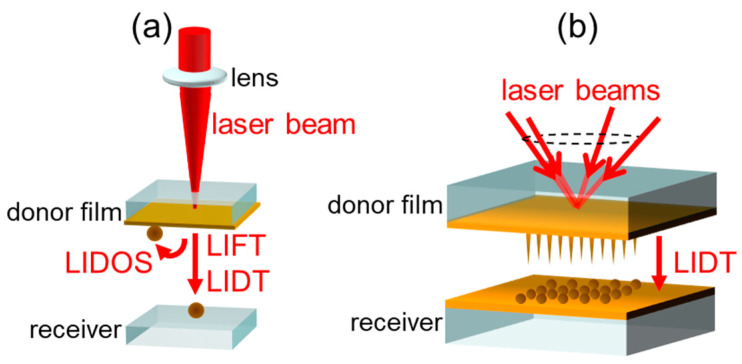
(**a**) Schematic illustration of laser-induced forward transfer (LIFT), laser-induced dot transfer (LIDT), and the laser-induced dot caught on source target (LIDOS) [18] process. (**b**) LIDT with interference pattern [17].

The first laser-induced forward transfer (LIFT) technique was examined in 1970 [1]. It was called as “laser typewriter”, in which an ink ribbon was used as a source target. In this technique the irradiated region is transferred to the receiver substrate, as shown in Figure 1a. The target thin films were extended to various materials such as metals [2], dielectrics [3], biomaterials [4], and fluorophore [5], and microscopic process measurement techniques using image laser spectroscopy have been applied to investigate the LIFT process [6,7]. The laser-induced dot transfer (LIDT) technique, a type of LIFT, was developed for depositing nanodots which are smaller than laser wavelengths [8,9,10,11]. The mechanism involves the liquid behavior of solute metals and their freezing, called the solid–liquid–solid (SLS) mechanism [12]. Here, SLS enables the fabrication of a variety of nanostructures other than nanodots using LIDT, such as the nanobump [13], nanodrop [13,14], nanowhisker [12,15], nanocrown [15,16], etc. Furthermore, we have investigated interference laser processing for two decades. In these studies, the aligned nanostructures mentioned above have been successfully fabricated in a single shot of laser irradiation. We applied this technique to LIDT as shown in Figure 1b, and aligned Au nanodots with Λ=3.6 μm square matrices were successfully deposited [17].

In this study, we used Pt thin film as a source target for LIDT combined with the interference laser processing technique, and the nanodots deposited on the receiver substrate were measured by scanning electron microscopy (SEM). The size distributions were analyzed on parameters such as pulse energy. The results that we obtained are supported by a heuristic model.

## 2. Materials and Methods

**Figure 2:**
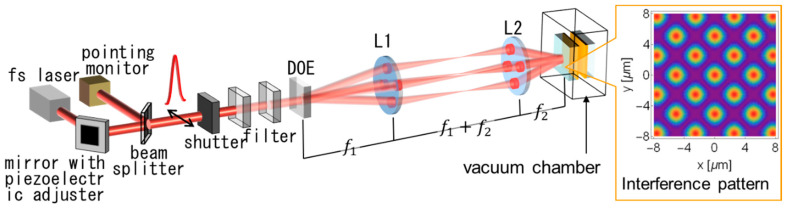
Experimental setup. Right inset shows the simulation result of the interference pattern on the irradiation plane. fs laser: femtosecond laser; DOE: diffractive optical element; L1 and L2: achromatic lenses.

The experimental setup is shown in Figure 2. A laser operated at a 785 nm center wavelength with a 240 fs pulse width was used. The pointing was stabilized with a piezo-actuated mirror by <10 μrad h^−1^. The beam was split by a diffractive optical element (DOE) into four 1st order diffracted beams. They were correlated and formed an interference pattern on the backside of the donor Pt thin film through a de-magnification system consisting of two achromatic convex lenses ($f_{1}=200$ nm, $f_{2}=50$ nm). The two lenses and the zero-order beam were placed coaxially with the DOE perpendicular to the axis, and at the same time they were properly spaced. The verticality was set by aligning the direction of the return light to the light source. The donor substrate was placed with mechanical precision so that it was perpendicular to the axis. In this way, an interference pattern was formed on the donor substrate. The zero-order beam was dumped between the lenses. The right inset in Figure 2 is a simulation result of the interference pattern [19], and the period was Λ=3.6 μm. The 50 nm thick Pt film deposited onto a 1mm thick silica glass substrate that was used as a donor film target. Here, in a quote from a previous paper, the thickness of the donor Au film was 40 nm. An Au receiver film 100 nm thick deposited onto a 1mm thick silica glass substrate which was placed in contact facing the donor film. The LIDT experiment was performed in a vacuum chamber (P<1.3 kPa). The off-line image analysis explained in Section 3.2 was performed using ImageJ (NIH).

## 3. Results and Discussion

### 3.1. SEM Images of the Pt Nanodots

**Figure 3:**
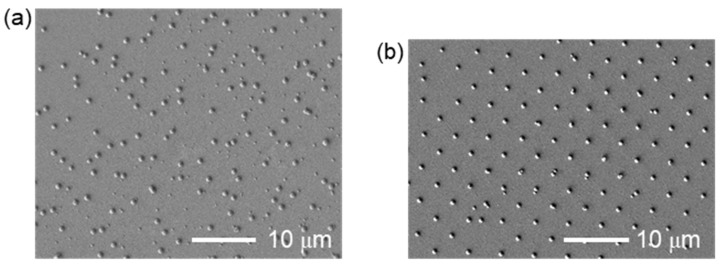
Scanning electron microscopy (SEM) images of (**a**) Pt nanodots and (**b**) Au nanodots deposited by LIDT with the interference pattern of a fs laser. The original image of (**b**) is the same as in [17] Nakata et al. 2020. Int. J. Exrem. Manuf. 2, 025101.1-5. CC-BY-3.0.

Typical SEM images of the Pt and Au nanodots [17] are shown in Figure 3. The surface morphology was measured by a scanning electron microscope (JSM-7400FS, JEOL, Tokyo, Japan). When $F_{peak}$ was the peak fluence, the area with a fluence higher than $F_{peak}/e^{2}$ was 0.073 mm^2^. The pulse energy for the Pt and Au target was 69 and 97 μJ, and the average fluence ($1/e^{2}$) in the area was 94.2 and 133 mJ/cm^2^, respectively. It is apparent that the Pt and Au nanodots were transferred successfully. On the other hand, the Pt nanodots are deposited in a dispersed position, though the Au nanodots are in array corresponding to the interference pattern which is shown in the right inset in Figure 2. This tendency did not change with the fluence, and small nanodots are found between the large nanodots in the case of Pt.

**Figure 4:**
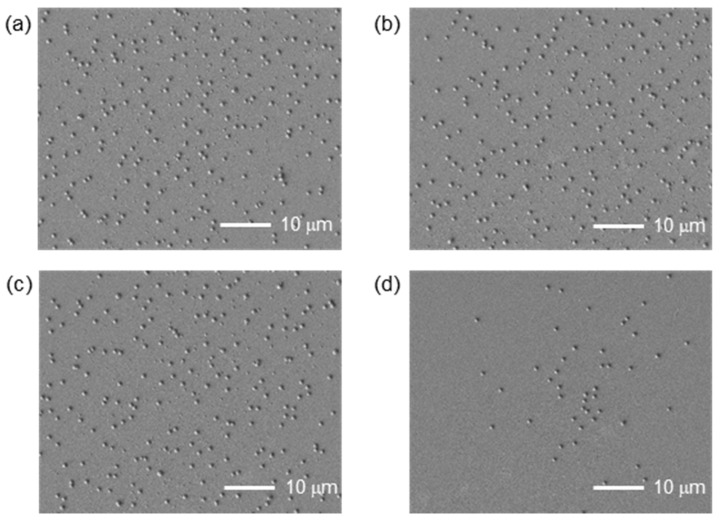
SEM images of Pt nanodots with different averaged fluences. (**a**) 106 mJ/cm^2^, (**b**) 94 mJ/cm^2^, (**c**) 84 mJ/cm^2^, (**d**) 75 mJ/cm^2^. (**b**) shows the same area as in Figure 3a, but is slightly wider.

The SEM images of Pt nanodots as a function of the averaged fluence are summarized in Figure 4. The measurement range is located approximately at the center of the processed area. At a lower fluence of 75 mJ/cm^2^, the number of nanodots is small. It seems that the size of the nanodots appears to be classified into two types at a higher fluence, the statistical analysis of which is explained in the following Section 3.3.

**Figure 5:**
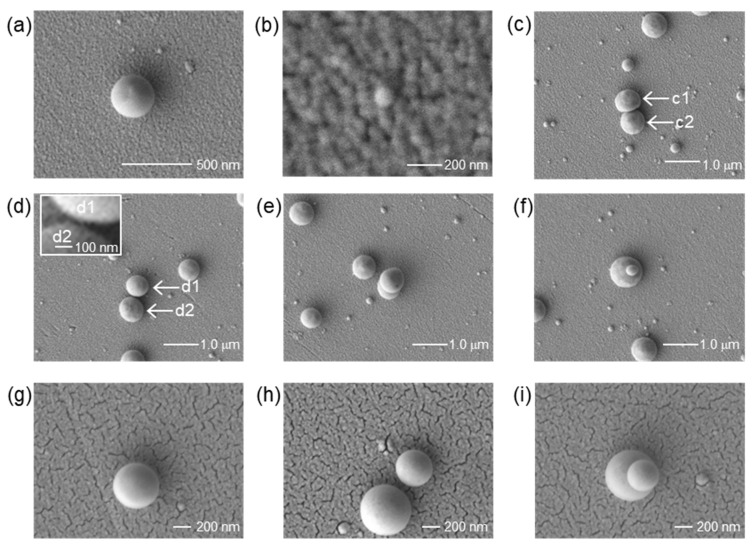
SEM images of the nanodots. (**a**,**b**) Pt solo nanodots, (**c**) Pt adjoining nanodots, (**d**) Pt nanodots with gap, and (**e**,**f**) Pt stacking nanodots. (**g**,**h**) and (**i**) are solo, adjoining and stacking Au nanodots, respectively. The pixel size of the image is (**b**) 2.36 nm/pixel, (**g**–**i**) 1.88 nm/pixel, and 4.7 nm/pixel otherwise. Therefore, the first digit of the size in the text is for reference.

In the previous experiment using Au donor film, a variety of unit structures such as solo, adjoining, and stacking nanodots were observed [17]. We found similar unit structures in this experiment, as shown in Figure 5. Here, all images are of structures fabricated under the fluence of 106 mJ/cm^2^, but similar structures were seen in the other fluences of 94 or 84 mJ/cm^2^. As mentioned below, the size of the nanodots are classified into two types. Figure 5a is a representative large nanodot, which has circular shape with a diameter of D=627 nm. Here, the circularity $f_{circ}$ and roundness $f_{r}$ of the nanodot are defined by the following equations:

(1) $$f_{circ}=4\pi S/P^{2}$$

(2) $$f_{r}=4S/{\left(2a\right)}^{2},$$

where S is the surface area of the nanodot, P is the perimeter, and 2a is the length of the major axis, assuming the shape to be an ellipsoid. For this nanodot, we obtained $f_{circ}=0.90$ and $f_{r}=0.98$, so it is a fairly round circle. The three-dimensional shape is considered to be a collapsed sphere, as in previous experiments using Au thin film [17]. On the other hand, a representative small nanodot is shown in Figure 5b. The diameter is D=125 nm. Such fine nanodots were seen in the entire measurement area at all fluences.

Similar to the case of Au donor film, we found solo, adjoining, and stacking nanodots, as shown in Figure 5c–f. Here, Figure 5g–i are images of Au nanodots [17]. In the case of the adjoining nanodots that are in contact as shown in Figure 5c, the shape of the c1 nanodot is squashed. It is an ellipse with a minor axis of 600 nm and a major axis of 746 nm. This implies that at the timing of deposition near c2, c1 was at high temperature and soft. The diameter of the c2 nanodot is 707 nm. Whereas, Figure 5d shows nanodots with a nanogap. Both d1 and d2 are circular with diameters of D=621 and 702 nm, respectively. The gap length is Δl=46 nm.

In contrast to the case with Au donor film, the number of stacking nanodots, which are shown in Figure 5e,f, seemed to be quite small. The mechanism will be discussed in Section 3.3.

### 3.2. Statistical Analysis

**Figure 6:**
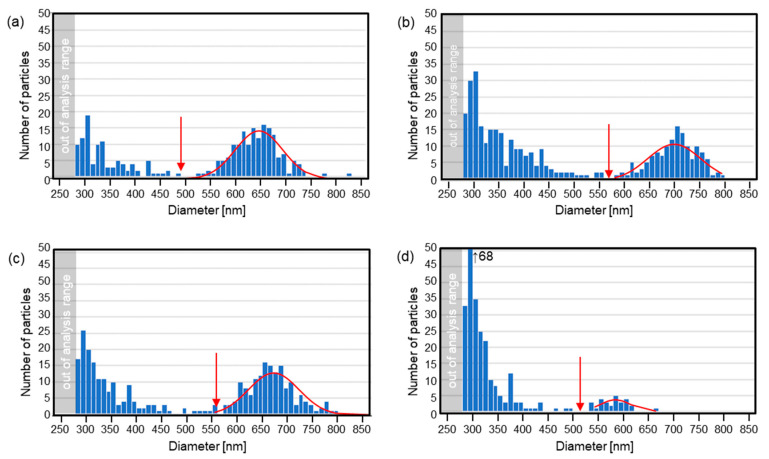
Nanodot size distributions. The averaged fluence was (**a**) 106, (**b**) 94, (**c**) 84, and (**d**) 75 mJ/cm^2^, respectively. Alphabetical numerals correspond to those in Figure 4. The red curve on each graph corresponds to a normal distribution fit on the data set starting from the red arrows to the right in each case.

**Figure 7:**
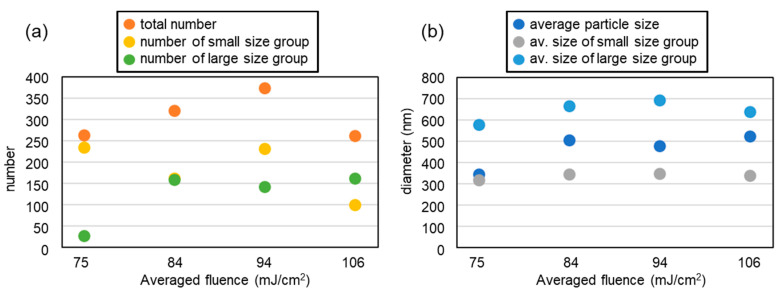
(**a**) nanodot number and (**b**) size distributions. The average fluence was 106, 94, 84, and 75 mJ/cm^2^, respectively.

In a past LIDT experiment, Dr. Narazaki reported that single or multi nanodot ejection occurs from a focal spot of a laser [8]. The same phenomena was observed in the case of LIDT using an interference pattern with an Au film target [17]. In this subsection, nanodot size distribution is analyzed from the images in Figure 4. Here, adjoining and stacking nanodots are excluded from the calculation. The minimum size measurement limit due to the resolution of the image is approximately D=280 nm. Figure 6 shows the nanodot size distribution as a function of the averaged fluence. In all conditions, the distribution is split into two parts: small dot and large dot groups, as divided by the red arrows. The total number of each group is summarized in Table 1, and plotted in Figure 7a. Here, the number of the spots in the corresponding area shown in the images in Figure 4 is 223. So, it is interesting that the number of nanodots is always larger than the number of ablation spots in the interference pattern. This directly proves that multiple nanodots can be generated from a single spot.

At the lowest fluence, the number of large dots is low, though that of small dots is high. On the other hand, many large nanodots are formed at higher fluences. The total number in of nanodots in the large group does not change much above 84 mJ/cm^2^. The red curve on each graph corresponds to a normal distribution fit on the data set starting from the red arrows to the right in each case. The averaged diameter of the large nanodot group is largest at around 84 to 94 mJ/cm^2^, and decreased at the highest fluence, as shown in Figure 7b. In addition, the number of the small nanodot group decreased at the highest fluence. These results suggest that the vaporized metal dissipated or was deposited as a thin film on the receiver substrate without condensation at the highest fluence. Here, it should be noted that there is a possibility of the nanodots’ shapes being squashed, depending on the temperature at which the particles are deposited, which is likely to vary with the average fluence and the thermal properties of the receiver substrate.

Here, the shape and diameter of the nanodots fabricated by various methods is compared. The circularity and roundness are excellent in most of the LIDT experiments including this paper. On the other hand, the size distribution was one group in the previous experiment, but it is divided into two groups in this experiment. The large nanodot group has an average size of about 580 to 690 nm, which is comparable to the previous interference LIDT experiment using Au thin film, and other group’s single LIDT experimental results [8,20]. On the other hand, the nanodots fabricated using LIDOS with an Au thin film, in which nanodots are deposited on a source target placed in air as seen in Figure 1a, have a smaller average particle size of less than 200 nm. This is probably due to the fact that the interference pattern period is 1.93 μμm, which is about half that of the present study [18]. It is important to note that for precise comparison, it is necessary to prepare the same experimental systems. The smallest standard deviation in LIDOS was 3 nm [18], which is far smaller than the values shown in Table 1. With even larger spot sizes, higher pulse energies, and thicker film targets, the deposit becomes a micron-sized dot or larger diameter film structure, and this process is called LIFT [1,2,3,4,5,21]. In the case of pulsed-laser deposition (PLD), micron-sized droplets are ejected from the ablation spot [22]. In this technique, some tens of nm-sized particles are formed due to the condensation at high atmospheric gas pressure conditions [23,24]. Here, chemosynthesis is useful to fabricate a number of nm-sized nanodots and nanorods. In summary, utilizing LIDT with an interference pattern is a good alternative method to PLD. In addition, there is still a possibility that the array structure and uniform size distribution of Pt nanodots can be achieved by optimizing the parameters. We will discuss this in the next section.

### 3.3. Heuristic Model of LIDT

**Figure 8:**
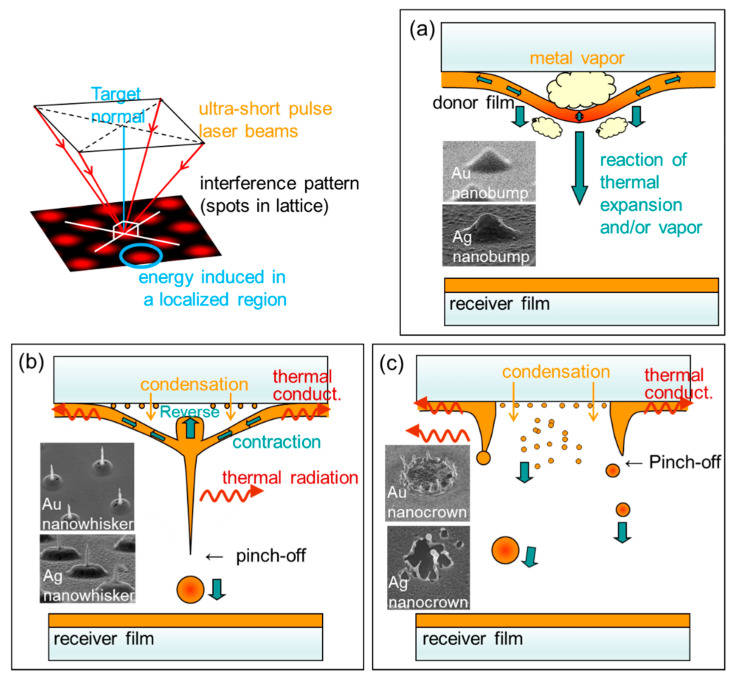
Schematic explanation of solid–liquid–solid (SLS) process in LIDT. The upper left sketch is a schematic of four-beam interference, in which the bright spots are in lattice. (**a**) induction of energy into a localized region results in a partial liquid motion of solute metal thin film by the reaction due to the thermal expansion of the film or vapor pressure. (**b**) partial liquid motion results in the simultaneous formation of a nanodot and a nanowhisker. (**c**) nanodots are ejected in a splashy manner. Insets in each illustration are copied from our past experiments [12,13,15,16].

In this subsection, the formation mechanism of nanodots is discussed based on the above observations and previous experiments. Figure 8 provides a schematic explanation of the mechanisms of LIDT via SLS, which has been discussed based on the experimental results of the interference laser processing. The upper left figure illustrates the interference laser processing. Here, we focus on a spot in the interference pattern. In the past experiment with Au and Ag thin film targets, we have selectively fabricated metal nanowhiskers [12,15] and nanocrowns [15,16], as shown in the inserted pictures in Figure 8b,c, respectively. Usually, nanowhiskers and nanodrops [13,25] are formed as shown in Figure 8b, but nanocrowns are formed as shown in Figure 8c when the film thickness is relatively thin or the interference pattern has a wider period, i.e., when the spot size is relatively large [15,16]. In the former case, a nanodot is formed by surface tension and deposited onto the receiver substrate, which was shown in the previous paper using Au donor film [17]. In this case, the position of the nanodots and the interference pattern are in good agreement. In addition, as seen in the water droplet behavior [26,27,28], the formation of a second and subsequent nanodots may occur, which are deposited as the neighbor of the first nanodots [8,17]. Thus, the film thickness and spot size have been considered to be the key parameters for determining either of the processes explained in Figure 8b,c, up to this point.

On the other hand, in this experiment, only by changing the thin film material to Pt did we find a dispersion in the deposition position and the generation of a greater number of nanodots than the number of spots. Here, three general mechanisms of nanodot formation can be considered: formation at the spot center by surface tension of the liquid metal, formation at the edge of the nanocrown, and condensation of the metal vapor. Here, the last mechanism does not occur in this experiment because it only occurs under high pressure. As shown in Figure 4, the nanodots are not aligned according to the interference pattern. In addition, the stacking nanodots in this experiment, which are shown in Figure 5e,f, were not frequently found which may be due to the fact that major nanodots are ejected in a splashy manner, not sequentially from the same spot [26,27,28]. These suggest the ejection of nanodots from the nanocrowns. On the other hand, the sizes are divided into two groups, as shown in Figure 6. The average size of the large sized group is similar to the previous results of the LIDT using Au, where an ablation and a Au nanodot corresponded one-to-one. Additionally, their number is smaller than that of the number of ablation spots. Thus, it is possible that the nanodots in the larger size group were formed by the process explained in Figure 8b, and the smaller group nanodots were formed by the process explained in Figure 8c. In addition, the most reliable way to confirm the type of LIDT mechanism is to measure the donor thin film after LIDT with SEM. Recovery of the donor thin film substrate after the process is possible by improving the method of fixing the donor thin film target, which will be done in the future. Furthermore, imaging measurement of the LIDT process is also an alternative. In past experiments, the resolution of the microscopic imaging laser-induced fluorescence (2D-LIF) [6] or scattering imaging technique [7] were suited to measure the behavior of the micron-sized ejecta in LIFT. On the other hand, the very small size of the nanodots in this LIDT setup makes it difficult to measure them with these techniques.

Here, we discuss on the cause of the difference in results by comparing the experimental conditions with the previous experiment where Au donor thin film was used, and the period of the interference pattern was the same, 3.6 μm. The film thickness and averaged fluence agree within a difference of 20 to 30%. In addition, the melting points of Au and Pt are 1064 and 1768 °C, respectively. The surface tensions at those temperatures are 907 and 1784 dyn/cm respectively [29], and the latter is almost twice as large. The viscosity is between 3.7 and 4.9 cP at 1333–1640 °C in the case of liquid Au [30], and is 6.74 cP at 1768 °C in the case of liquid Pt [31]. At present, it is not clear which physical properties are the key parameters to govern the process. Here, molecular dynamic simulation will be a powerful tool to investigate the underlying mechanisms and key parameters [32]. There is still the possibility of achieving an array structure and uniform size distribution of the Pt nanodots by optimizing the parameters, and further experiments are needed.

## 4. Conclusions

The interference pattern of a femtosecond laser was applied to the LIDT technique, and a number of nanodots with diameters of some hundreds of nm with good roundness and circularity were fabricated. A typical nanodot had a circularity $f_{circ}=0.90$ and roundness $f_{r}=0.98$. A comparison of the number of nanodots produced and the number of processed spots in the interference pattern revealed that multiple nanodots were formed from a single spot. The size distribution was divided into two groups, and the ratio was affected by the average fluence. The smallest average diameter of the large nanodot group was 581 nm with a smallest standard deviation of 30 nm at 75 mJ/cm^2^.

In terms of applications, Pt is useful as a catalyst. Its size is relatively larger than that of a typical catalyst [33] and so the combined use of plasmonic interactions is promising. Compared with bottom-up methods such as chemosynthesis, SLS can fabricate pure nanodots because no catalyst or chemosynthetic solutions are required. It requires no further processes, such as rinsing or annealing. The size distribution of Pt nanodots is reasonably uniform, but the processing parameters for fabricating aligned nanodots needs to be further explored. These advantages will broaden the range of applications in nano-3D printing and catalyst and plasmonic applications.

## Figures and Tables

**Table 1:** Results of the analysis of particle size distribution: number, mean size, standard deviation, and values for each group divided by size (indicated by red arrow in Figure 6). Alphabetical numerals correspond to those in Figure 4 and Figure 7.

Averaged Fluence (mJ/cm^2^)	(a) 106	(b) 94	(c) 84	(d) 75
(a-1) total number	263	375	322	264
(a-2) number of small size group	100	233	162	236
(a-3) number of large size group	163	142	160	28
(b-1) average particle size	526	480	506	347
(b-2) average size of small size group	339	349	345	319
(b-3) average size of large size group	641	698	668	581
(c-1) standard deviation (SD)	151	176	171	88
(c-2) SD of small size group	48	58	62	36
(c-3) SD of large size group	46	52	50	30
